# 19734 L-type calcium channels in cerebellar neuron development and motor learning

**DOI:** 10.1017/cts.2021.424

**Published:** 2021-03-30

**Authors:** DeAnna O’Quinn, Aislinn Williams, Ashley Parker, Bryn Myers, Ashley Plumb, Hsiang Wen, Marisol Lauffer

**Affiliations:** University of Iowa Institute for Clinical and Translational Science

## Abstract

ABSTRACT IMPACT: We aim to understand how LTCCs impact cerebellar function. OBJECTIVES/GOALS: L-type calcium channels (LTCCs) are important in activity-dependent neurite outgrowth, which comprises neurite initiation and elongation. We used cerebellar granule neurons (CGNs) to differentiate between LTCC effects on neurite initiation vs elongation. We also tested cerebellar function in mice lacking specific LTCCs with behavioral assays. METHODS/STUDY POPULATION: CGNs were cultured from 129SvEv mouse pups at P4-P6. Potassum chloride (50mM) was used to stimulate neuronal cultures for 24 hours. Isradipine (20nM) was added to culture medium to inhibit all LTCCs for 1 hour. For Cav1.2 deletion, we crossed Cav1.2 conditional knockout mice (Cav1.2-cKO) to Syn-Cre mice (for deletion in most neurons) or Atoh1-Cre mice (for deletion in CGNs). The Cav1.2-cKO line was maintained on a 129SvEv background. For constitutive Cav1.3 deletion, mice were maintained on a C57BL/6NTac. Behavioral tasks included open field, rotarod, and Erasmus Ladder. Data were analyzed with sexes combined and separated to assess for sex as a biological variable. Studies were analyzed by one-way ANOVA, two-way ANOVA, or generalized linear mixed model, where appropriate. RESULTS/ANTICIPATED RESULTS: CGNs exhibited an increase in neurite initiation but not elongation when stimulated with potassium chloride, consistent with previous reports of activity-dependent neurite outgrowth in this cell type. LTCC inhibition with isradipine blunted KCl-induced neurite initiation. We observed no change in the length of either primary or secondary neurites with isradipine treatment with or without KCl stimulation. In our behavioral experiments, we observed no deficits in open field, rotarod, or Erasmus Ladder when Cav1.2 was deleted in most neurons (driven by Syn-Cre expression) or in cerebellar granule neurons (driven by Atoh1-Cre expression). In contrast, loss of Cav1.3 was associated with impaired motor learning in the rotarod task without evidence of ataxia on Erasmus Ladder. DISCUSSION/SIGNIFICANCE OF FINDINGS: We show a specific role for LTCCs in activity-dependent CGN neurite initiation. While loss of Cav1.2 does not affect motor learning, loss of Cav1.3 does impair motor learning. Our results help expand our understanding of LTCC function in cerebellar neurodevelopment and function.

